# Crash testing machine learning force fields for molecules, materials, and interfaces: molecular dynamics in the TEA challenge 2023†

**DOI:** 10.1039/d4sc06530a

**Published:** 2025-02-03

**Authors:** Igor Poltavsky, Mirela Puleva, Anton Charkin-Gorbulin, Grégory Fonseca, Ilyes Batatia, Nicholas J. Browning, Stefan Chmiela, Mengnan Cui, J. Thorben Frank, Stefan Heinen, Bing Huang, Silvan Käser, Adil Kabylda, Danish Khan, Carolin Müller, Alastair J. A. Price, Kai Riedmiller, Kai Töpfer, Tsz Wai Ko, Markus Meuwly, Matthias Rupp, Gábor Csányi, O. Anatole von Lilienfeld, Johannes T. Margraf, Klaus-Robert Müller, Alexandre Tkatchenko

**Affiliations:** a Department of Physics and Materials Science, University of Luxembourg L-1511 Luxembourg Luxembourg alexandre.tkatchenko@uni.lu igor.poltavskyi@uni.lu; b Institute for Advanced Studies, University of Luxembourg Campus Belval L-4365 Esch-sur-Alzette Luxembourg; c Laboratory for Chemistry of Novel Materials, University of Mons B-7000 Mons Belgium; d Department of Engineering, University of Cambridge Trumpington Street Cambridge CB2 1PZ UK; e Swiss National Supercomputing Centre (CSCS) 6900 Lugano Switzerland; f Machine Learning Group, Technical University Berlin Berlin Germany; g BIFOLD, Berlin Institute for the Foundations of Learning and Data Berlin Germany; h Fritz-Haber-Institut der Max-Planck-Gesellschaft Berlin Germany; i Vector Institute for Artificial Intelligence Toronto ON M5S 1M1 Canada; j Wuhan University, Department of Chemistry and Molecular Sciences 430072 Wuhan China; k Department of Chemistry, University of Basel Klingelbergstrasse 80 CH-4056 Basel Switzerland; l Chemical Physics Theory Group, Department of Chemistry, University of Toronto St. George Campus Toronto ON Canada; m Friedrich-Alexander-Universität Erlangen-Nürnberg, Computer-Chemistry-Center Nägelsbachstraße 25 91052 Erlangen Germany; n Department of Chemistry, University of Toronto St. George campus Toronto ON Canada; o Acceleration Consortium, University of Toronto 80 St George St Toronto ON M5S 3H6 Canada; p Department of NanoEngineering, University of California San Diego 9500 Gilman Dr, Mail Code 0448 La Jolla CA 92093-0448 USA; q Heidelberg Institute for Theoretical Studies Heidelberg Germany; r Luxembourg Institute of Science and Technology (LIST) L-4362 Esch-sur-Alzette Luxembourg; s Department of Materials Science and Engineering, University of Toronto St. George campus Toronto ON Canada; t Department of Physics, University of Toronto, St. George campus Toronto ON Canada; u University of Bayreuth, Bavarian Center for Battery Technology (BayBatt) Bayreuth Germany; v Department of Artificial Intelligence, Korea University Seoul South Korea; w Max Planck Institut für Informatik Saarbrücken Germany; x Google DeepMind Berlin Germany

## Abstract

We present the second part of the rigorous evaluation of modern machine learning force fields (MLFFs) within the TEA Challenge 2023. This study provides an in-depth analysis of the performance of MACE, SO3krates, sGDML, SOAP/GAP, and FCHL19* in modeling molecules, molecule-surface interfaces, and periodic materials. We compare observables obtained from molecular dynamics (MD) simulations using different MLFFs under identical conditions. Where applicable, density-functional theory (DFT) or experiment serves as a reference to reliably assess the performance of the ML models. In the absence of DFT benchmarks, we conduct a comparative analysis based on results from various MLFF architectures. Our findings indicate that, at the current stage of MLFF development, the choice of ML model is in the hands of the practitioner. When a problem falls within the scope of a given MLFF architecture, the resulting simulations exhibit weak dependency on the specific architecture used. Instead, emphasis should be placed on developing complete, reliable, and representative training datasets. Nonetheless, long-range noncovalent interactions remain challenging for all MLFF models, necessitating special caution in simulations of physical systems where such interactions are prominent, such as molecule-surface interfaces. The findings presented here reflect the state of MLFF models as of October 2023.

## Abstract 2

We present a comprehensive analysis of the capabilities of modern machine learning force fields to simulate long-term molecular dynamics at near-ambient conditions for molecules, molecule-surface interfaces, and materials within TEA Challenge 2023.

## Graphical abstract

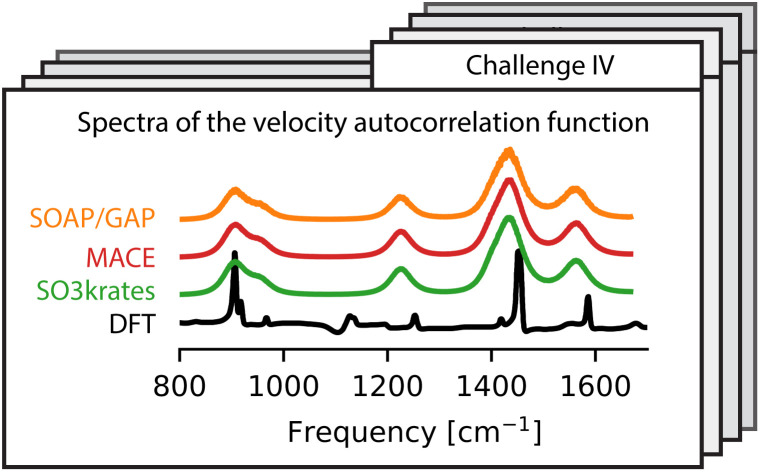

## 1 Introduction

The practical application of machine learning force fields (MLFF) aims to enhance the capabilities of computational chemistry reference methods, enabling dynamical simulations that would otherwise be unfeasible. Achieving this goal requires a high degree of trust in simulation results, allowing MLFF models to become standard tools in research and industry pipelines. While architecture development is greatly facilitated by easy “pointwise” testing of models on standardised train/test splits of toy problems, and this approach has been standard in the machine learning (ML) community, creating models that are actually useful for materials and molecular science research requires complicated system-specific evaluation. Even the earliest successful models in the materials field that were targeting specific systems already did this, demonstrating high accuracy in computing observables such as phonon spectra, phase transitions, defect formation energies, *etc.*, as well as pointwise accuracy of reproducing the potential-energy surfaces and atomic forces.^1–9^ Later, as the computer science community engaged with the problem of molecular modeling, their practices also came to be prominent and, especially in works that compared different ML architectures, developers assessed the accuracy of models mostly by evaluating errors in energies and forces relative to the ground truth they were targeting.^10–29^ There is a widely held view that we need to return to assessing observables.^30^ In the meantime, sophisticated MLFF accuracy measures^31–35^ and visualization tools^29,36^ have been developed to address ML models' performance on local and global measures. It was suggested in particular that long molecular dynamics (MD) simulations^19,37–49^ provide a robust test of MLFF reliability as predictors of physical behavior when mean absolute error (MAE) or root mean square error (RMSE) may be insufficient or even misleading when considered on their own.^50–57^

In this study, we evaluate the quality of modern MLFF architectures by comparing the outcomes of MD simulations performed with MACE,^13,14^ SO3krates,^22,23^ sGDML,^17,18,38,58^ SOAP/GAP,^2,6^ and FCHL19*^11,12,59,60^ models. MACE and SO3krates are equivariant message-passing graph neural networks (NNs), representing many-body information about the geometric atomic configuration employing spherical harmonics and radial distributions function learned through multilayer perceptrons. SO3krates also relies on an equivariant attention mechanism to enhance the model's efficiency. FCHL19*, sGDML, and SOAP/GAP are kernel-based ML architectures. FCHL19* and SOAP/GAP are based on local atom-centered representations, while sGDML employs a global descriptor. Full details of the MLFFs are available in the ESI of ref. 61. We would like to highlight that the MLFF architectures included in the TEA Challenge 2023 were limited to those whose developers could participate in the benchmark. This approach was adopted to minimize the risk of misinterpretation or drawing misleading conclusions due to potential mistraining. The complete list of modern MLFFs is considerably broader. Prominent MLFFs that did not participate include ANI,^62^ Alegro,^24^ ACE,^63^ ALIGNN-FF,^64^ AIMNet2,^65^ DeepMD,^66,67^ Elemental-SDNNFF,^68^ FIREANN,^69^ FLARE,^70^ G-MBNN,^71^ GPTFF,^72^ MTP,^73^ NequIP,^25^ PIP,^74,75^ SevenNet,^76^ SNAP,^77^ and M3GNet,^78^ among others. To ensure that the current benchmark can be extended to include any additional MLFF model in the future, all the data and scripts necessary to train other ML architectures and replicate the simulations and analyses are available online and upon request.

Given the computational expense of explicit electronic structure methods, often the only plausible test for the performance of an ML model (apart from comparing to experimental observables, which brings its own complexities) is another model trained and used under identical conditions. Achieving consistency in results across different ML architectures would mark a significant milestone in the development of MLFFs. This work builds upon our previous manuscript, “Crash Testing Machine Learning Force Fields for Molecules, Materials, and Interfaces: Model Analysis in the TEA Challenge 2023,” which provided a detailed analysis of force and energy predictions on test datasets.^61^ In contrast, this study focuses on analyzing various observables derived from MD simulations. Only trajectories that provide sufficient statistical data under the specified simulation conditions are considered. Each challenge is independently analyzed to assess the ability of different MLFF architectures to simulate specific types of systems. We focus on evaluating the capability of MLFFs to perform classical MD simulations under ambient and near-ambient conditions. While these simulations cover a broad range of potential MLFF applications, they do not address all possible scenarios. For example, simulations that capture nuclear quantum effects, such as imaginary-time path-integral MD,^79^ or those involving chemical bond breaking, fall outside the scope of this study. Tackling such challenges would require additional components, including advanced sampling techniques to capture low-probability or classically forbidden system geometries, as well as costly multi-reference *ab initio* calculations to accurately describe dissociation processes. Each of these represents an open challenge in its own right.

This article is divided into four sections as follows. In Section 2, we present a comprehensive analysis of the classical MD simulations conducted for each system studied in the TEA Challenge 2023. These systems include two biomolecular systems (alanine tetrapeptide and *N*-acetylphenylalanyl-pentaalanyl-lysine), a 1,8-naphthyridine/graphene interface, and a methylammonium lead iodide perovskite. The starting point of each of the 12 MD trajectories simulated in each experiment and the corresponding MLFF models can be found in the Zenodo archive https://doi.org/10.5281/zenodo.13832724 with trajectories available upon request. From their analyses, we extract key insights into the applicability and reliability of MLFFs, and identify opportunities for further development and improvement. Section 3 contains guidelines for the development, training, and application of MLFFs. Section 4 represents the overall conclusions.

## 2 Results and discussion

### 2.1 Alanine tetrapeptide

We begin our analysis of the MD trajectories provided by different MLFF models with the simplest case among the TEA 2023 challenges: the alanine tetrapeptide molecule in Challenge I. For a decade, organic molecules with a few dozen atoms in the gas phase have been routinely treated with different MLFF architectures. Additionally, such molecules are well within the capabilities of DFT codes, which can routinely compute hundreds of thousands of geometries without excessive computational effort. In this particular case, the reference dataset is comprised of 85 109 molecular geometries representing an *ab initio* NVT MD trajectory generated at 500 K (taken from MD22 benchmark,^58^ details available in Section 2 of ref. 61). This comprehensive dataset facilitated the generation of three distinct training sets, categorized by the distance between the farthest non-hydrogen atoms, which served as a measure of molecular compactness: (1) the complete set, encompassing representative samples of both extended and compact Ac-Ala3-NHMe structures; (2) the folded set, consisting of the 70% most compact structures extracted from the MD trajectory; and (3) the unfolded set, comprising the remaining 30% of less compact structures from the same trajectory. Details of the training procedures for different MLFFs for this and the other Challenges are available in ESI of ref. 61.

**Fig. 1:**
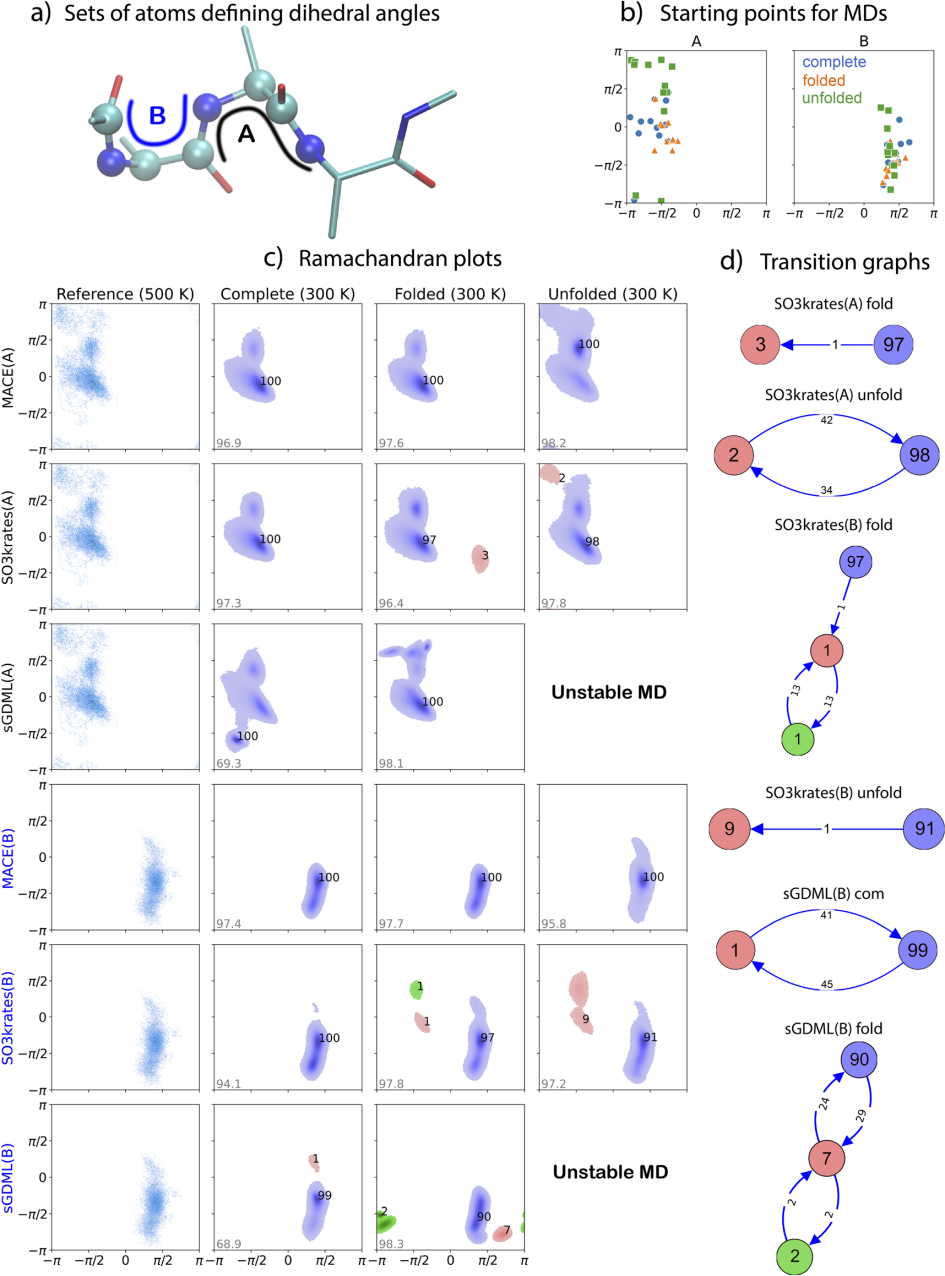
Challenge I, Alanine tetrapeptide: analysis and Ramachandran plots for MD simulations at 300 K. (a) Diagram of the peptide depicts the peptide with atom sets A and B forming two consecutive pairs of dihedral angles (left four atoms – *x*-axis, right four atoms – *y*-axis). (b) Initial points for 12 MD trajectories. (c) Ramachandran plots for the reference systems at 500 K and for MD simulations at 300 K using MACE, SO3krates, and sGDML MLFFs trained on com (complete), fold (folded), and unfold (unfolded) datasets. The numbers near the clusters indicate their relative population (in percent), while the grey number in the lower left corner of each plot shows the percentage of configurations from the MD trajectories identified as belonging to one of the clusters. (d) Graphical representation of the transitions between different (meta)stable domains. The values on the arrows show the number of transitions identified in the dynamics.

Analysis of the MD trajectories and a comparison between the outputs for the different MLFFs are presented here *via* Ramachandran plots, as shown in Fig. 1. The Ramachandran plots are useful for visualizing the allowed conformational space of the peptide backbone.^80^ For the Ac-Ala3-NHMe tetrapeptide, two selected pairs of dihedral angles in Fig. 1a, A and B, are conventionally referred to as *ϕ*_2_/*ψ*_2_ and *ϕ*_1_/*ψ*_1_, respectively.^80–82^ The initial points for MD simulations are depicted in Fig. 1b.

In order to obtain an informative picture of the results, analysis involving a clustering algorithm was carried out to identify the high density regions of population during the MD and filter out noise and unrepresentative low density areas.^83,84^ The full step-by-step algorithm description is available in the ESI.† Our analysis algorithm identifies different (meta)stable domains in the Ramachandran plots, illustrated in various colors, Fig. 1c. The transitions between these (meta)stable domains obtained with SO3krates folded/unfolded and sGDML complete/folded models are represented in the graph form in Fig. 1d. It is important to note that the benchmark reference trajectory was obtained at 500 K to allow for more extensive sampling of the conformational space and bond lengths. To ensure statistically significant results, only the Ramachandran plots for ML models that produced stable 1 ns dynamics are presented. Consequently, the analyses are based on MLFF MD trajectories obtained at 300 K, as most MLFF models failed to produce stable 1 ns dynamics at 500 K. Nevertheless, the Ramachandran plots for the reference *ab inito* MD at 500 K still provide a qualitative guideline for the 300 K MLFF MD results.

*The MACE models trained on both complete and folded datasets exhibit excellent mutual agreement*. However, they undersample the upper left corner of the Ramachandran plot for A and the upper part of the dihedral cluster for B compared to the reference data. Both of these areas correspond to the highly unfolded conformations of the tetrapeptide. Training the MACE model on the unfolded dataset results in better qualitative agreement with the reference MD trajectory. Quantitative agreement estimation is challenging due to the short length of the *ab initio* MD trajectory and the difference in temperatures.

*The SO3krates models display distinct Ramachandran profiles depending on whether they are trained on complete, folded, or unfolded datasets*. Firstly, when trained on both complete and folded datasets, SO3krates model also undersample regions of highly unfolded conformations, similar to MACE. This indicates that this might be due to lower simulation temperature compared to the reference. Notably, the SO3krates model trained on the folded dataset exhibits additional metastable states with low populations (3% for A and 1% for B) and low transition probabilities. For dihedral B, the SO3krates model trained on the unfolded dataset identifies an extra metastable region with a 9% cluster population, though the transition probability into this state is low, with only one transition observed in 12 ns of total dynamics (12 × 1 ns). The seeds of this cluster also appear as two small clusters with a 1% population and relatively high mutual transition rates in the SO3krates model trained on the folded dataset. These molecular geometries were not observed in MD simulations using the SO3krates model trained on the complete dataset or by any MACE or sGDML models. However, exploration to these regions have been studied previously in the original SO3krates article.^23^ It is worth noting that the two small clusters in the *SO3 fold* plot, dihedrals B, and the two clusters in the *SO3 unfold* plot, dihedrals A, can be merged due to their relatively high transition rates. The clustering algorithm employed here uses a predefined fixed number of transitions to merge clusters chosen to suit the data in general. It does not account for their population, providing suboptimal results when at least one of the clusters is small.

*The sGDML model trained on a complete dataset also demonstrates acceptable results*. The total number of transitions between the two clusters identified for B is 86, slightly below the manually selected threshold of 100 for merging the clusters into one. However, *removing parts of the reference geometries in folded or unfolded datasets leads to significant differences in the Ramachandran profiles or even MD instability*. This sensitivity is attributed to the sGDML model's interpolation in the space of a global system descriptor of inverse distances, making the model highly dependent on the quality and completeness of the training dataset compared to MLFFs employing local descriptors.

In ESI, a comprehensive Table SI 1† lists the chemical bonds responsible for the instability of all MLFF architectures trained on different datasets at 300, 500, and 700 K. Most broken bonds involve carbon atoms connected to other elements. Additionally, there are notable differences in bond-breaking patterns between kernel-based and equivariant NN-based ML models. For sGDML, SOAP/GAP, and FCHL19*, the specific bond causing molecular instability was readily identifiable. By contrast, for SO3krates, bond breaking exhibited an explosion-like behavior, with a large part of the molecule decomposing into atoms within a few dozen steps, making it challenging to pinpoint the exact bond responsible for the instability.

In summary, when trained on a complete dataset, we observed strong mutual agreement in the molecular dynamics of the alanine tetrapeptide generated by MACE and SO3krates models. Discrepancies arise when the training datasets lack either folded or unfolded geometries. Both models reasonably explored the unfolded potential energy surface (PES) basin when trained on the unfolded dataset. The MACE folded model is consistent with the complete one. The SO3krates folded and unfolded models were mostly consistent, while the sGDML MLFFs demonstrated increased sensitivity to variations in the training data.

### 2.2 *N*-Acetylphenylalanyl-pentaalanyl-lysine

**Fig. 2:**
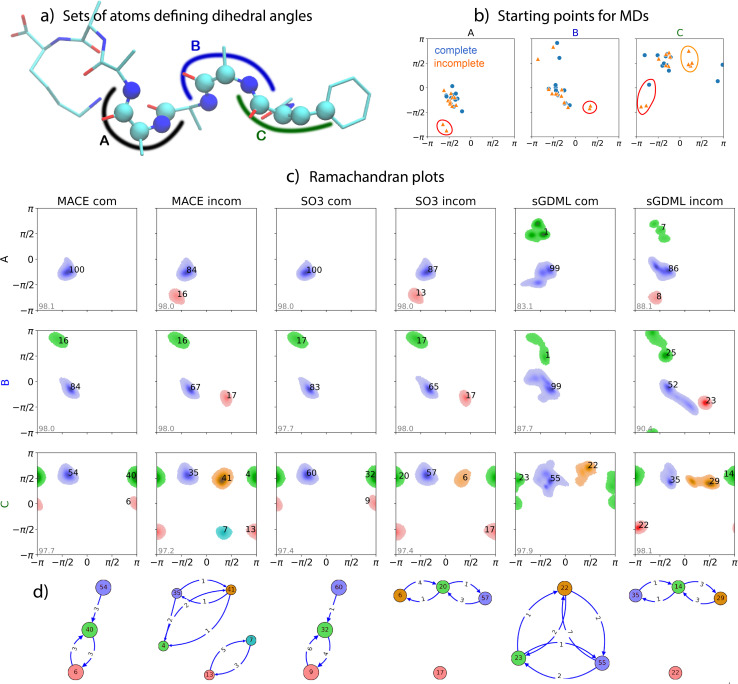
Challenge II, *N*-acetylphenylalanyl-pentaalanyl-lysine peptide: Analysis and Ramachandran plots for MD simulations at 300 K. (a) Diagram of the peptide with sets of atoms A, B, and C forming three consecutive pairs of dihedral angles (right four atoms – *x*-axis, left four atoms – *y*-axis). (b) Initial points for 12 MD trajectories. (c) Ramachandran plots for MD simulations employed different MLFF models: MACE com (complete), MACE incom (incomplete), SO3 com, SO3 incom, sGDML com, and sGDML incom. The numbers near the clusters indicate their relative population (in percent). The grey number in the bottom left corner of each plot shows the relative number of configurations (in percent) from the MD trajectories identified as belonging to one of the clusters. (d) Graphical representation of transitions between different (meta)stable domains for dihedrals C. The values on the arrows show the number of transitions identified in the dynamics.

The second challenge in the TEA Challenge 2023 was performed on a larger organic system, namely the protonated Ac-Phe-Ala5-Lys peptide. The dataset, comprising 100 000 reference geometries with energies and forces calculated using the PBE0 (ref. 85) exchange-correlation functional and the nonlocal MBD (MBD-NL)^86^ method for modeling dispersion interactions, was specifically generated for the TEA Challenge 2023. Full details can be found in Section 2 of ref. 61. The main aim of the challenge was to assess the ability of MLFFs to handle different types of reference data incompleteness. To this end, two training datasets were created for the Ac-Phe-Ala5-Lys peptide system based on MD simulations around the 200 lowest energy conformers. The “complete” dataset consisted of 20 randomly selected molecular configurations from each of these trajectories, while the “incomplete” dataset contained 32 randomly selected configurations from only 125 trajectories. The clustering algorithm used here is the same one as for Challenge I (full details in ESI†). Fig. 2 presents the Ramachandran plots for three selected pairs of neighboring dihedral angles along the peptide. The dihedral angle pairs, labeled as A, B, and C (see Fig. 2a), represent key cases for comparing different MLFFs. The initial points for MD simulations are shown in Fig. 2b. Only the Ramachandran plots for ML models that produced stable 1 ns long dynamics are displayed. The FCHL19* and SOAP/GAP MLFFs did not achieve the required 1 ns to perform statistically-significant analysis. Our analysis identifies distinct (meta)stable domains in the Ramachandran plots, represented in different colors, Fig. 2c. Since the original dataset was not derived from an MD trajectory, reference density plots are unavailable. Transition graphs illustrate the transitions between (meta)stable domains for dihedrals C across different ML models, Fig. 2d.

We begin by comparing the MD results of the MACE and SO3krates models, each trained on complete (com) and incomplete (incom) datasets, Fig. 2c. The most noticeable difference is the increased number of clusters in the dynamics generated by the models trained on the incomplete dataset, primarily reflecting variations in the starting points for the MD simulations. Additional red clusters appear for the A and B dihedrals due to distinct starting points in the incomplete trajectories (highlighted by red circles in Fig. 2b). These starting points significantly differ from other initial configurations, leading to divergences in the Ramachandran plots. Both models, however, accurately reproduce the close-to-equilibrium regions of the PES for A and B, providing similar positions of the minima and shapes of the probability distributions around them (within 3% agreement in population distributions between clusters).

For the C dihedrals, a block of four starting points (highlighted by an orange circle in Fig. 2b) appears like an orange cluster. Additionally, a shift in the red cluster position from 0 to –π/2 along the *Y*-axis is attributed to differences in starting configurations between the complete and incomplete datasets (emphasized by a red circle in Fig. 2b). Differences in the MLFFs from the MACE and SO3krates architectures significantly influence the distribution of the C dihedrals, as supported by transition graphs in Fig. 2d, differing especially in the case of training on an incomplete dataset. The MACE MLFF shows a 54% relative population for the complete C dihedral case for the largest cluster (–π/2, π/2) compared to 60% with the SO3krates MLFF. This difference stems from a higher transition probability within the MACE PES from this state to the green cluster (–π, π/2). Conversely, the green cluster population is higher with the MACE PES at 40%, compared to 32% for the SO3krates PES, due to a nearly twice higher transition probability between the green and red (π, 0) metastable states with the SO3krates MLFF trained on the complete dataset.

These results suggest that *the primary difference between MACE and SO3krates MLFFs lies in the description of out-of-equilibrium regions of the PES*, which are responsible for rare transitions or large geometry fluctuations. This is also underpinned by the PES analyses provided in the first part of the manuscript, see Fig. 5, of ref. 61 or the MD results of Challenge III presented later in this article. Further comparison of the MACE and SO3krates models trained on the incomplete dataset for the C dihedrals supports this. Noticeably different transition patterns emerge, as well as differing cluster populations and even the appearance of an extra metastable state (blue cluster) within the PES reconstructed with the MACE MLFF (incomplete dataset). It is also likely that such differences would emerge between different MLFFs trained on the same data with the same architecture, just using a different set of initial weights. We want to state, though, that the statistically converged analyses of the transition patterns would require more extended MD simulations or the employment of enhanced sampling techniques which is beyond the scope of the current work. Simultaneously, the MACE and SO3krates architectures, when trained on the complete dataset, demonstrate remarkable mutual agreement in modeling the dynamics of the Ac-Phe-Ala5-Lys peptide. This consistency underscores the capability of modern MLFFs based on equivariant NN with different architectures to handle relatively large and complex organic molecules and model their dynamics and transitions.

For the sGDML architecture, we observe only qualitative agreement with the results from both NNs in Challenge II. The shape and population of clusters differ significantly due to the global nature of the sGDML model, which requires a reliable and comprehensive set of representative query configurations for effective interpolation between system states. Achieving such a representative set is challenging for large molecules undergoing complex structural transformations, especially with a relatively small training dataset of 4000 configurations. Consequently, the *sGDML model operates in a low-data regime, leading to a notable depreciation in performance*.

In ESI, Table SI 2† details the broken chemical bonds responsible for the instability of MD simulations across all MLFF architectures. We observe behavior similar to that reported for Challenge I. Notably, the kernel-based MLFFs utilizing local descriptors, namely SOAP/GAP and FCHL19*, could not sustain stable dynamics over a 1 ns duration for both peptides evaluated in this study. This finding indicates that MD simulations employing global sGDML models or NN architectures, incorporating nonlocality through message-passing elements, exhibit greater stability than MLFFs based solely on local ML models.

In summary, our observations indicate that the results for MACE, SO3krates, and sGDML MLFFs in this test were consistent with those from Challenge I. Overall, we can conclude that equivariant NN MLFFs can reliably reproduce the dynamics of organic molecules (at least up to 100 atoms). The main discrepancies between MD results occur when the training datasets lack representative reference geometries. This suggests that active learning or similar iterative approaches^87–89^ should become an integral part of MLFF training procedures to ensure the completeness and representativeness of training datasets.

### 2.3 1,8-Naphthyridine molecule on graphene

**Fig. 3:**
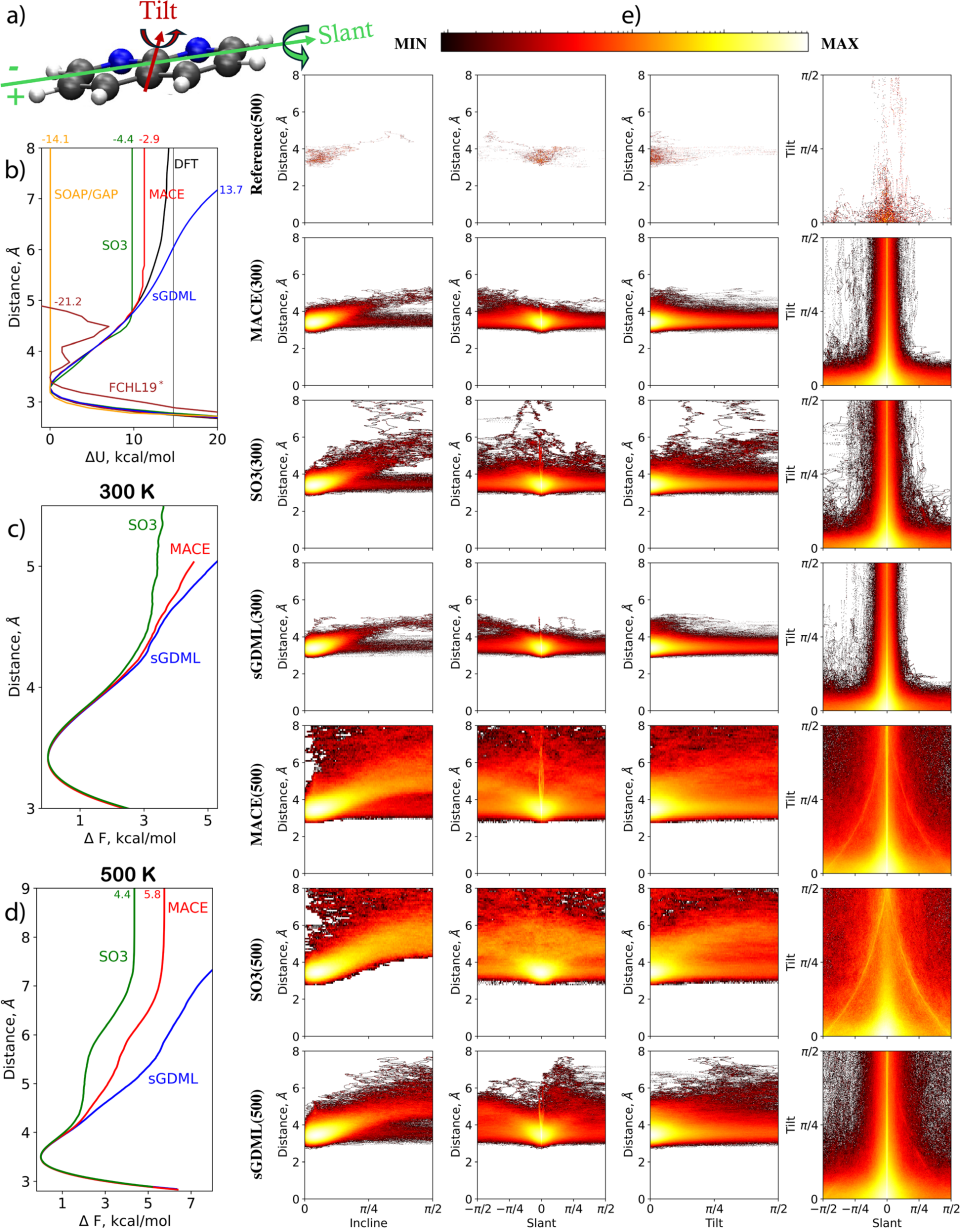
Analysis of MDs for 1,8-Naphthyridine molecule adsorbed on graphene. (a) Schematic representation of the molecule and definitions of tilt and slant angles. (b) Interaction energy profile (Δ*U*) as a function of the distance between the centers of mass of the molecule and graphene. (c) and (d) Free energy change profile from MD simulations at 300 and 500 K, respectively. (e) Distributions of incline, tilt, and slant angles at different molecule-to-surface distances, and the tilt-slant angle distribution in MD simulations at 300 and 500 K using MACE, SO3krates, and sGDML models compared to the reference dataset.

For modern MLFFs, dealing with either a 1,8-Naphthyridine molecule or a pristine graphene sheet separately is easy. However, combining the two systems introduces additional complexity, namely the molecule–substrate interaction responsible for multiple key properties of the system. MLFFs have to learn this interaction simultaneously with the much stronger covalent bonds in 1,8-naphthyridine and graphene. In our analyses, the focus is on four measures that capture the molecule–substrate interaction. Firstly, the distribution of the *distance D* between the graphene sheet and the molecule, defined as a distance between the averaged graphene plane (AGP) and the center of mass of the molecule, is considered. Secondly, the *incline* angle between the molecule and the surface, defined as the angle between the averaged plane of the molecule (H atoms excluded) and the AGP is analysed. Lastly, the *tilt* and *slant* angles are investigated. See plots of all the interaction's aspects in Fig. 3. Note that the molecule is symmetrical for the tilt rotation (the tilt angle is defined in the range [0, π/2]), while it is asymmetric for the slant rotation (the slant angle is defined in the range [–π/2, π/2]). Fig. 3 illustrates the results for MLFF MD simulations conducted at 300 and 500 K. Notably, all ML models encounter angles and distances that extend significantly beyond those observed in shorter reference MD simulations at 500 K, indicating that the MLFF models operate in an extrapolation regime.

*At 300 K, sGDML, SO3krates, and MACE produced similar patterns for all four examined dependencies*. This includes subtle features found in agreement between models observed in the plot of incline angle probability as a function of surface-to-molecule distance. A notable split for angles close to π/2 (molecular orientation perpendicular to the graphene surface) is visible for sGDML, SO3krates, and MACE. The split arises from the asymmetry in the molecule's slant rotation, where the side containing N atoms can approach the surface closer than the sides with H atoms attached to C atoms. The molecular asymmetry also explains the trend to negative slant values in the distance *versus* slant angle plots. Furthermore, it is the reason for the asymmetric large fluctuations of the slant angle in the tilt *versus* slant angle plots – a negative slant angle indicates that the side of the molecule with N atoms is closer to the surface. The main disagreement between the models is a tendency for the molecule to desorb from the surface when SO3krates models is employed. This tendency is absent in the MD simulations produced with the MACE and sGDML MLFFs.

*At 500 K, the predictions between the MLFFs begin to diverge*. The MD simulations obtained with MACE and SO3krates models differ drastically from those produced by sGDML. Both the MACE and SO3krates models predict the molecule's desorption from the surface, while the sGDML model keeps the molecule within an 8 Å distance. This suggests that the PES profile for the molecule in the direction perpendicular to the graphene surface predicted by sGDML differs from that predicted by the NNs. To verify this conjecture, the molecule-surface interaction energy is computed as a function of the distance, see Fig. 3b. The computation starts from the relaxed structure (obtained using the reference DFT setup), and confirmations are produced, for which the z-coordinates of the atoms belonging to the molecule are moved further from or closer to the surface in 0.1 Å steps. This provides us with the Δ*U*(*D*) dependence computed at the reference level of accuracy. The same calculations are repeated using the MACE, SO3krates, sGDML, SOAP/GAP, and FCHL19* models. Notably, some MLFFs failed to provide reasonable energy predictions at large molecule-to-surface distances. Therefore, the minimum energy within the distance range of 3 to 4 Å is chosen for each method as the zero energy level. The thin black line in Fig. 3b corresponds to the DFT energy at an infinite molecule-to-surface separation.

In complete agreement with the MD simulations, the *SO3krates and MACE models significantly underestimate the potential energy minima compared to the DFT reference*. Fig. 3b shows the difference between the DFT and MLFFs energy predictions at an 8 Å distance as small numbers on the top. Notably, this distance is beyond the cutoff for both NNs, leading them to predict zero interaction between the molecule and the surface. The MACE model underestimates the molecule-surface interaction energy at 8 Å by 2.9 kcal mol^−1^ and SO3krates by 4.4 kcal mol^−1^. For the adsorption minimum, when aligning the DFT and MLFFs energies at infinite distance, the MACE and SO3krates models underestimate the value by 3.5 and 4.9 kcal mol^−1^, respectively.

Interestingly, *this underestimation* of the molecule-surface interaction minimum *aligns with the high accuracy in predicting the force acting on the molecule as a whole in the test set*. The reference molecule-to-surface distances are limited to an interval of 3 to 5 Å. Within this range, the curvature of the Δ*U*(*D*) function obtained from DFT calculations and those predicted by MACE and SO3krates models agree. Moreover, these distances fall within the cutoff radii for MACE and SO3krates, which are 6 Å and 5 Å, respectively. This results in minor MAEs in forces, 0.12 kcal (mol^−1^ Å^−1^) for MACE and 0.58 kcal (mol^−1^ Å^−1^) for SO3krates, despite a significant underestimation of the barrier.

Fig. 3c and d illustrate the differences of free energies of adsorption calculated employing different MLFFs. The free energies derived from MD trajectories *via* the thermodynamic integration method,^90^ are significantly smaller than the adsorption energy estimations calculated as the difference between the energy of the DFT-optimized structure and the state, for which the molecule and the surface are at infinite separation. This discrepancy arises due to the substantial rotational freedom of the molecule's plane relative to the surface plane. Specifically, the free energy minima predicted by the MACE and SO3krates models at 500 K are only 5.8 and 4.4 kcal mol^−1^, respectively, whereas the adsorption energies predicted by these models are 11.3 and 9.8 kcal mol^−1^.

*The kernel-based models also face significant challenges in reproducing the molecule-surface interaction*. The sGDML model over-stabilizes the system due to an artificial barrier at intermediate distances. For instance, at 8 Å, the model overestimates the DFT energy by 13.6 kcal mol^−1^. The artificial barrier only disappears at much larger distances, above 30 Å. The SOAP/GAP model fails to reproduce any molecule-to-surface attraction, providing a flat interaction profile immediately after the expected physisorption minima around 3.2 Å. The behavior of Δ*U*(*D*) within the FCHL19* MLFF lies somewhere between SOAP/GAP and the NNs. Qualitatively, the model reproduces the repulsion and a part of the attraction regions but with additional peculiar minima and the largest error at 8 Å distance of 21.2 kcal mol^−1^ among all MLFFs participating in the TEA Challenge 2023.

A significant point is that adding more training points with a better sampling of molecule-to-surface distances can only help the global sGDML MLFF to improve the description of the molecule-surface interaction. The finite cutoffs intrinsic to all other MLFFs participating in TEA 2023 would prevent them from correctly describing the molecule adsorption process or long-term dynamics unless additional elements targeting long-range interactions are incorporated into these MLFFs. We would like to emphasize that the above statement pertains specifically to the versions of the MLFF models that participated in the TEA Challenge 2023. The updated version of SO3krates (the pre-trained SO3LR model^91^ for bio-molecular simulations includes long-range electrostatic and universal dispersion interactions) includes the option to incorporate long-range interactions. With the appropriate reference data, SO3LR could accurately describe the adsorption process.

In ESI, Table SI 3† details the broken chemical bonds for the instability of MD simulations across all MLFF architectures.

In summary, molecule–surface interfaces need careful attention when using MLFFs for simulations. Modern MLFFs achieve high accuracy, capturing detailed system behavior with MAEs and RMSEs in the fraction of one kcal mol^−1^ and kcal (mol^−1^ Å^−1^). However, they require embedding long-range interaction models into MLFF architectures to handle system elements beyond the intrinsic cutoff distances, enhancing simulation accuracy and reliability.

### 2.4 Methylammonium lead iodide perovskite

The periodic structure of MAPbI_3_ features an organic methylammonium (MA) cation at the center of the inorganic eight corner-sharing [PbI_6_]_4_-octahedral. The stability of the simulations was assessed by applying two different thresholds for the organic and inorganic parts of the structure: 2 and 4.3 Å, respectively. The organic threshold was defined using the covalent radius. In contrast, the inorganic threshold was based on experimental results from various scattering techniques for the radial distribution function of the Pb–I ionic bond.^92^

**Fig. 4:**
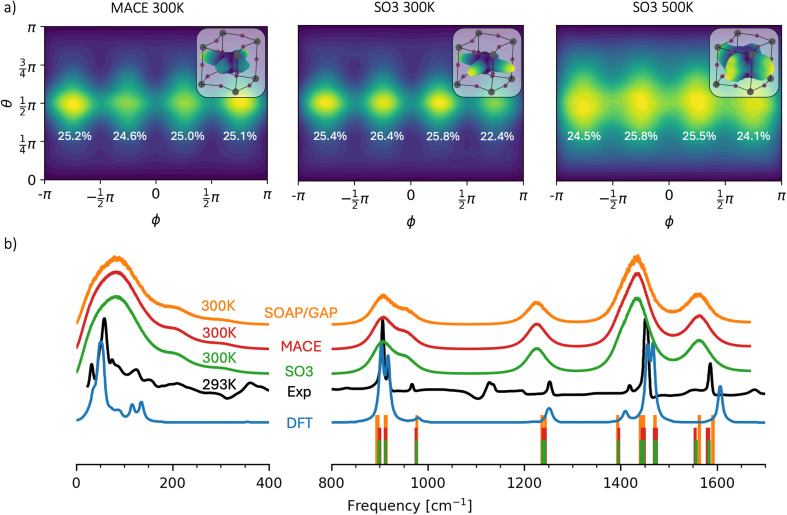
Observables extracted from the MD simulations of the orthorhombic phase of MAPbI_3_. (a) Distribution of C–N bond orientation in spherical coordinates. The numbers near the maxima indicate the participation ratios of the bond orientation for four different regions of *ϕ*. 3D spatial representations of the distribution within the PbI framework on a logarithmic scale are shown in the upper right corners. (b) Spectral analysis of the velocity autocorrelation function (solid lines) obtained from MD simulations using SO3krates (green), MACE (red), and SOAP/GAP (orange) models at 300 K, compared to the experimental infrared spectrum^93^ (black) and DFT within the harmonic approximation^93^ (blue). The vertical bars indicate phonon frequencies calculated within the harmonic approximation for each MLFF model, spanning a frequency range of 800 cm^−1^ to 1800 cm^−1^.

MA cations within the PbI framework are characterized by ionic interactions with the rest of the lattice. To investigate how ML models reproduce these weak interactions, we computed the distribution of the orientation of the C–N bond of MA cations. The results in spherical coordinates are shown in Fig. 4a for the MACE and SO3krates models in cases where MD simulations produced sufficiently long (over 10 ns) trajectories. The polar angle *θ* is the angle between the *z*-axis and the C–N bond, while the azimuthal angle *ϕ* is the angle between the orthogonal projection of the bond on the *x*–*y* plane and the *x*-axis. The upper right corner of the distribution plot for each set of MDs presents a 3-dimensional representation of the C–N orientation on a log scale for peak visibility. The numbers near the maxima of the distributions indicate the probability of the bond orientation for four regions: −π < *ϕ* < −π/2, −π/2 < *ϕ* < 0, 0 < *ϕ* < π/2 and π/2 < *ϕ* < π. The bond tends to orient into cavities in the PbI framework along the *x* and *y* axes for all presented MDs. As expected, the distribution for SO3krates at 500 K is more smeared than that for both NNs at 300 K. The unlikely orientation of the bond along the *z*-axis relates to the alignment of this axis with the *c*-axis of the orthorhombic structure of MAPbI_3_.

We would like to briefly address the stability issues encountered with the MACE model during MD simulations at 500 K. The MACE model permitted significant fluctuations in the positions of Pb atoms, which ultimately compromised the structural integrity of the system when these atoms approached the MA molecule. This instability can be attributed to the inadequate sampling of large atomic fluctuations in the reference dataset, which was derived from relatively short *ab initio* MD simulations.^94^ Additionally, the training dataset lacked supplementary data necessary for the ML models to capture these large-scale atomic movements accurately. Notably, the escape events involving Pb atoms in our simulations occurred after significantly longer times compared to the reference dynamics. Despite this, the SO3krates model demonstrated a markedly improved stability. In our tests, SO3krates resulted in only 2 out of 12 failed 1 ns long trajectories, compared to 12 out of 12 failures with MACE. However, it is important to note that SO3krates also encountered similar issues twice, underscoring the current limitations in accurately describing interatomic interactions at distances beyond the typical covalent bond range in a sparse training data regime. A comparative analysis of atomic radial distribution functions from MACE and SO3krates MD simulations at 500 K, alongside 1 ps *ab initio* MD results at the same temperature, is provided in ESI.†

Fig. 4b presents the spectral analysis of the MAPbI_3_ system for MACE, SO3krates, and SOAP/GAP models. This analysis yields the vibrational frequencies of the system, derived through the Fourier transform of the velocity autocorrelation function (VAF), which provides insights into vibrational dynamics. The experimental infrared (IR) spectrum and a DFT-simulated IR spectrum (within the harmonic approximation) are also shown for reference. Since IR intensities are governed by changes in dipole moments, while VAF spectra are based on velocity correlations, the resulting peaks from the VAF method are generally broader, with different intensity ratios. Nevertheless, the VAF-based frequency positions can still be compared meaningfully to the IR spectra. Unlike the harmonic approximation assumed in DFT, the VAF approach can recover anharmonic vibrational modes. For instance, a notable feature at 200 cm^−1^ is observed in both the MD-derived and experimental IR spectra but is absent from the DFT predictions. Overall, *all MLFF models produce nearly identical spectra, showing good agreement with experimental data and DFT* in terms of peak positions.

At the same time, *the experimental peaks* observed at 360 and 1140 cm^−1^*were not detected by either MLFF*. We attribute this discrepancy to the *limitations of the reference DFT calculations*. Notably, these peaks are also absent in the DFT spectra presented in Fig. 4. However, it is important to note that the DFT functionals used in this study differ from those in ref. 93. Unfortunately, computing the IR spectra for MAPbI_3_ with the unit cell size used for the TEA Challenge 2023 at the level of accuracy PBE + MBD-NL is not feasible.

Additionally, phonon frequencies, depicted as vertical bars on Fig. 4b, were calculated within the harmonic approximation for each model, covering the frequency range from 800 cm^−1^ to 1800 cm^−1^. To obtain these frequencies, the 2 × 2 × 2 MAPbI_3_ system was first optimized separately by each MLFF model using the BFGS algorithm implemented in ASE, with a convergence criterion set to a maximum force component of 0.005 eV Å^−1^.^95^ The phonon frequencies were then derived from these optimized structures using the finite difference method for each respective model, employing a 0.01 Å displacement. These frequencies are consistent across the models and align well with the IR spectra, demonstrating the MLFFs' ability to accurately capture the region of the PES near the system's equilibrium state. However, differences between the MACE, SO3krates, and SOAP/GAP models are slightly more pronounced in the harmonic spectra compared to the VAF spectra. This highlights the greater sensitivity of physical properties that depend on specific regions of the PES to the accuracy of the MLFFs, in contrast to more “global” statistical properties like VAFs, which reflect a broader portion of the PES.

In ESI, Table SI 4† details the broken chemical bonds manifesting in the instability of MD simulations across all MLFF architectures. The stability of MD simulations of MAPbI_3_ was primarily compromised by broken covalent bonds within MA cations, although C–N bond breaks were less frequent. Interestingly, the instabilities observed in the MD trajectories generated using the MACE model were triggered by significant fluctuations of the Pb atoms rather than covalent bond breakage.

In summary, kernel-based MLFFs failed to provide stable MD trajectories, with only the SOAP/GAP model successfully generating 2 out of 12 one ns-long trajectories without loss of structural integrity. In contrast, equivariant NNs demonstrated reliable stability and efficiency. Spectral analysis showed that MACE, SO3krates, and SOAP/GAP models aligned well with experimental and DFT-derived spectra, despite missing some peaks, likely due to limitations in the reference DFT calculations. Therefore, the main bottleneck for atomistic simulations in this class of systems (similar to organic molecules) is obtaining high-quality and representative reference data rather than the MLFF architectures themselves.

## 3 Guidelines for MLFF

The last two decades of developing MLFFs can be characterized as a rapidly growing research activity to create efficient, accurate, scalable, and transferable ML architectures. While this work continues and no architecture design has been universally accepted yet, other factors have become equally important. At the current level of accuracy achieved by modern MLFFs, the quality and completeness of training data and the training process have become defining factors. Below, we present a list of guidelines to follow in the development, training, and application of MLFFs.

(1) Cross-validation: even the most advanced single MLFF architecture should not be blindly trusted. Cross-checking results between different MLFF models can help to increase the reliability of simulations, particularly where reference data (computational or experimental) is sparse or unavailable.

(2) Detailed performance analysis: comparing MLFFs' performance based on overall MAE, RMSE, or similar aggregate measures are only sensible for simple and small systems with comprehensive datasets. In more complex cases, a detailed analysis of MLFF performance (per atom, per chemical element, per environment type) is crucial.

(3) Reducing heterogeneity of atomic errors: reducing the heterogeneity of atomistic MAEs while maintaining acceptable overall accuracy leads to more reliable MLFFs than those trained solely to minimize aggregated errors.

(4) Training dataset quality: the completeness and composition of training datasets significantly impacts MLFF performance. Using datasets that over-represent certain types of states can decrease overall MAE and RMSE but might lead to incorrect simulation results.

(5) Active learning: active learning and similar iterative techniques for correcting the training set should be intrinsic elements of the MLFF training process. Additionally, complementing the training dataset with structures corresponding to very small and very large interatomic distances, even if such situations are unlikely in expected application conditions, can improve MLFF quality, by enforcing the proper asymptotic behaviours.

(6) Incorporating multiscale forces: for systems with a multiscale composition (*e.g.*, atoms forming molecules, molecules forming molecular clusters), adding corresponding force terms into the MLFF loss function during training (with appropriate weights) can improve the reliability of system behavior during simulations.^96^ Minimizing only total atomistic errors might be insufficient and could lead to incorrect behavior of larger-scale system components.

(7) Appropriate accuracy levels: depending on the application, MLFFs with MAEs of, for instance, 0.5 or 0.1 kcal (mol^−1^ Å^−1^) might produce the same results in MD simulations. A more accurate model requires more computationally demanding reference data and is slower in production and training, without providing any significant practical benefits. Even within the same MLFF architecture, modellers should explore the tradeoff between model size, accuracy, and computational efficiency.

(8) Saving training information: it is crucial to document the complete training settings (hyperparameters), MLFF software version, and details of the training and validation datasets to ensure future applicability and potential retraining of an ML model. Ideally, this information should be automatically embedded in the MLFF model files, enabling the exact reproduction of the training process if the initial dataset is available.

(9) Transparency: developers of MLFFs should provide comprehensive details about modifications between different software and ML model versions, optimal preprocessing of training data beyond the intrinsic MLFF procedures, and any related offsets present in the outputs.

By adhering to these guidelines, the development and application of MLFFs can achieve greater reliability, ensuring more robust and trustworthy simulations.

## 4 Conclusions

The TEA Challenge 2023 extensively examined contemporary MLFF architectures, starting with error and stability assessments in the initial paper, “Crash Testing Machine Learning Force Fields for Molecules, Materials, and Interfaces: Model Analysis in the TEA Challenge 2023”. In this paper, we advance to a comprehensive comparative analysis of MD simulations conducted under identical conditions. Our objective is not to single out the best MLFF model but to present a current snapshot of the field, identifying reliable application areas and those requiring further improvement. This study focuses on three types of physical systems: organic molecules, molecule-surface interfaces, and 3D periodic systems.

For organic molecules, we observed excellent agreement between MD results obtained using MACE and SO3krates MLFFs when trained on comprehensive datasets. Discrepancies were primarily in the transition regions between (meta)stable states or large atomic fluctuations, likely due to the incompleteness of the training dataset rather than the ML architecture itself. The sGDML model also performed well for the smaller peptide, providing reliable MD trajectories. In contrast, the other two kernel-based ML models, SOAP/GAP and FCHL19*, exhibited insufficient stability, rendering them unsuitable for extended MD simulations.

Despite the success with MLFFs trained on comprehensive datasets, the dynamics of alanine tetrapeptide and *N*-acetylphenylalanyl-pentaalanyl-lysine molecules in Challenges I and II revealed noticeable artifacts when MLFFs were trained on incomplete datasets. This issue affected both kernel-based models and neural networks, underscoring the importance of reliable, high-quality, and comprehensive training data as a major bottleneck in developing effective MLFFs for organic molecules. Incorporating active learning or similar iterative approaches into MLFF training procedures is crucial to ensure thorough and representative datasets.

In Challenge III, which focused on studying the molecule-surface interface of a 1,8-Naphthyridine molecule on graphene, we identified significant limitations across all MLFFs participating in the TEA Challenge 2023. Although MLFFs have demonstrated strong performance in modeling covalent bonds, they currently lack the mechanisms needed to effectively capture long-range interactions. Consequently, none of the machine learning models were able to accurately reproduce the molecule desorption process, which can occur during extended MD simulations at virtually any temperature. Enhancing the reference dataset with more configurations that include larger molecule-to-surface distances would necessitate incorporating MLFF components that account for long-range non-covalent interactions. These mechanisms were absent in all but the sGDML MLFFs that participated in the TEA Challenge 2023. Nonetheless, addressing long-range non-covalent interactions remains a major focus for development, and by the time of the manuscript's publication, corresponding architectural elements had been proposed and implemented in some of the MLFFs. One should check the description of the relevant version of an MLFF software package.

Lastly, our evaluation of the 3D periodic system MAPbI_3_, kernel-based MLFFs struggled to maintain stable molecular dynamics trajectories without structural integrity loss. Conversely, MACE and SO3krates architectures provided stable and similar MD trajectories at 300 K, effectively sampling the part of the PES well-represented in the training dataset. Spectral analysis indicated good alignment of MACE, SO3krates, and SOAP/GAP models with experimental and DFT-derived spectra. Therefore, periodic systems like MAPbI_3_ can be considered within the reliable application range of modern MLFFs. The primary challenge for accurate atomistic simulations is once again obtaining high-quality and representative reference data. Additionally, optimizing models with respect to their model size to avoid unnecessary computational overhead is essential, especially in long-duration MD simulations of large systems, where achieving significant speedups is crucial.

## Abbreviations

SO3: SO3krates
sGDML: Symmetric Gradient Domain Machine Learning
SOAP/GAP: Smooth Overlap of Atomic Position/Gaussian Approximation Potential
FCHL19*: Faber–Christensen–Huang–Lilienfeld 19
MAE: Mean Absolute Error
RMSE: Root Mean Square Error
(in)com: (in)complete
(un)fold: (un)folded

## Data availability

All the TEA 2023 datasets are in the Zenodo archive https://doi.org/10.5281/zenodo.14138387.

## Author contributions

Igor Poltavsky – conceptualization, data curation, formal analysis, funding acquisition, methodology, project administration, software, supervision, validation, visualization, writing – original draft, writing – review & editing. Anton Charkin-Gorbulin – data curation, formal analysis, investigation, software, validation, visualization, writing – original draft, writing – review & editing. Mirela Puleva – data curation, formal analysis, investigation, project administration, software, validation, visualization, writing – original draft, writing – review & editing. Grégory Fonseca – data curation, formal analysis, investigation, software, validation, visualization, writing – original draft, writing – review & editing. Ilyes Batatia – data curation, formal analysis, methodology, software, writing – review & editing. Nicholas J. Browning – data curation, formal analysis, writing – review & editing. Stefan Chmiela – funding acquisition, supervision, writing – review & editing. Mengnan Cui – data curation, formal analysis, methodology, funding acquisition, writing – review & editing. J. Thorben Frank – data curation, formal analysis, funding acquisition, methodology, software, writing – review & editing. Stefan Heinen – data curation, formal analysis, methodology, software, writing – review & editing. Bing Huang – data curation, formal analysis, methodology, software, writing – review & editing. Silvan Käser – data curation, formal analysis, methodology, software, writing – review & editing. Adil Kabylda – data curation, formal analysis, methodology, software, writing – review & editing. Danish Khan – data curation, formal analysis, methodology, writing – review & editing. Carolin Müller – data curation, funding acquisition, supervision, writing – review & editing. Alastair J. A. Price – data curation, formal analysis, methodology, software, writing – review & editing. Kai Riedmiller – data curation, formal analysis, funding acquisition, writing – review & editing. Kai Töpfer – data curation, formal analysis, methodology, software, writing – review & editing. Tsz Wai Ko – writing – review & editing. Markus Meuwly – funding acquisition, resources, supervision, writing – review & editing. Matthias Rupp – data curation, writing – review & editing. Gabor Csanyi – funding acquisition, resources, supervision, writing – review & editing. O. Anatole von Lilienfeld – funding acquisition, resources, supervision, writing – review & editing. Johannes T. Margraf – funding acquisition, resources, supervision, writing – review & editing. Klaus-Robert Müller – funding acquisition, resources, supervision, writing – review & editing. Alexandre Tkatchenko – conceptualization, funding acquisition, methodology, project administration, resources, supervision, writing – review & editing.

## Conflicts of interest

GC has equity interest in Symmetric Group LLP that licenses force fields commercially and also in Ångström AI, Inc.

## Supplementary Material

SC-016-D4SC06530A-s001
